# Supplementary figures and images for: Depolarization of mouse DRG neurons by GABA does not translate into acute pain or hyperalgesia in healthy human volunteers
Source: PLoS One. 2024 Aug 26;19(8):e0307668. doi: 10.1371/journal.pone.0307668 (PMC11346724; doi:10.1371/journal.pone.0307668)

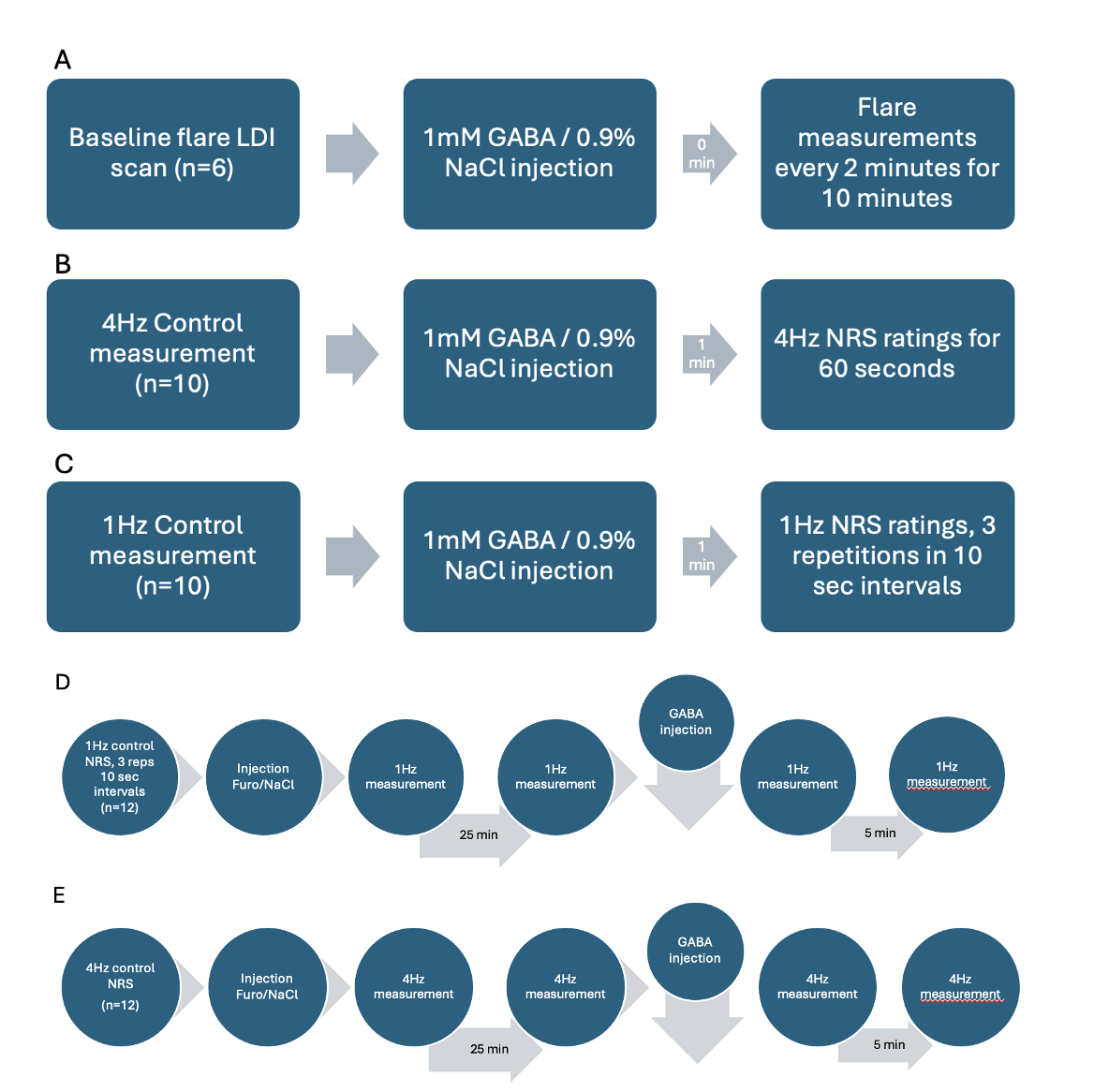

Supplement: S1 Fig — A) GABA or NaCl was injected into each respective forearm and axon-reflex flare (skin vasodilation) recorded using LDI for 10 minutes. Corresponding to Fig 2A. B) Electrically-evoked pain responses to 1Hz half period sinusoidal current and 4Hz sinusoidal current were assessed before and 1 minute after injection in verum and control treated forearms. Corresponding to Fig 2B. C) Furosemide (1mg) or NaCl were injected intradermally in either arm. Corresponding to Fig 2C. D) At baseline (prior to any injections) and 25 minutes after furosemide / NaCl injection, pain ratings in response to continuous 4Hz sinusoidal current and 1Hz half period sinusoidal current were assessed. Corresponding to Fig 2D. E) GABA (1mM) was injected at the same site of previous furosemide and NaCl injection (randomized forearms) and pain responses to 1Hz half period sinusoidal (1mA) and 4Hz sinusoidal (0.2mA) currents were again assessed at 1 minute and 5 minutes after GABA injection. Corresponding to Fig 2E. (TIF) [file pone.0307668.s001.tif]

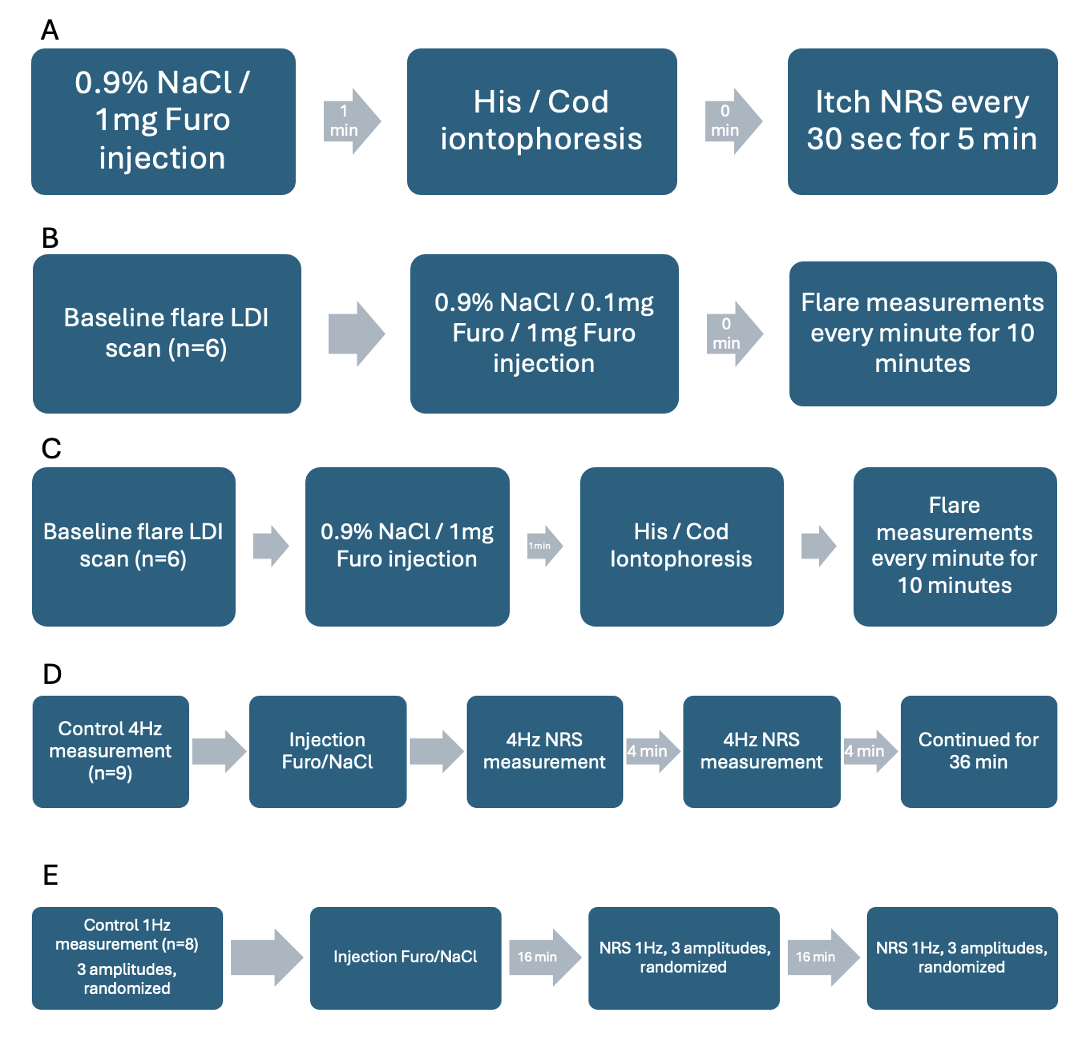

Supplement: S2 Fig — A) Skin sites approximately 10 cm distal to the cubital fossa on the left and right arms were injected with either 100μl furosemide or NaCl (randomized left or right). One minute after injection, histamine was applied by iontophoresis at the site of injection. Itch ratings (NRS) were recorded. Corresponding to Fig 3A. B) Skin sites approximately 10 cm distal to the cubital fossa on the left and right arms were injected with either 100μl furosemide at two different concentrations (0.1mg, light blue or 1mg, dark blue) or NaCl (0.9%). Skin vasodilation (LDI) was recorded. Corresponding to Fig 3B. C) One minute after injection of Furosemide or NaCl, histamine or codeine was applied by iontophoresis at the site of injection. Skin vasodilation (LDI) was recorded every minute for 10 minutes. 24 hours later, the same protocol was used for assessing the other (histamine or codeine) substance. D) Pain ratings (NRS) to sinusoidal current at 4Hz at an amplitude of 0.2mA delivered continuously for 1 minute before (baseline) and after intradermal injection of 1mg furosemide (blue diamonds) or 0.9% NaCl (black open circles), recorded every 4 minutes for 36 min (n = 9). E) Pain ratings (NRS) to 1Hz sinusoidal stimulation delivered at current intensities of 0.2, 0.6 and 1mA to the forearm skin sites before injection and 16 and 32 minutes after injection of 1mg Furosemide or NaCl. (TIF) [file pone.0307668.s002.tif]
