# Supplementary material for: Depolarization of mouse DRG neurons by GABA does not translate into acute pain or hyperalgesia in healthy human volunteers
Source: PLoS One. 2024 Aug 26;19(8):e0307668. doi: 10.1371/journal.pone.0307668 (PMC11346724; doi:10.1371/journal.pone.0307668)
Supplement: Attachment — Submitted filename: Response to Reviewers_.docx [file pone.0307668.s003.docx]

PONE-D-24-10034

Depolarization of mouse DRG neurons by GABA does not translate into acute pain or hyperalgesia in healthy human volunteers

PLOS ONE

Dear Dr. Schmelz,

Thank you for submitting your manuscript to PLOS ONE. After careful consideration, we feel that it has merit but does not fully meet PLOS ONE’s publication criteria as it currently stands. Therefore, we invite you to submit a revised version of the manuscript that addresses the points raised during the review process. It will be essential for you to respond to all of the reviewers' comments, but it is particularly important that you can successfully address: reviewer one's concerns about clarity/statistics and reviewer two's concern that the lack of effect of GABA could be due to the possibility that the chloride reversal potential is not above threshold for an AP (a significant consideration of this idea must be developed in the discussion).

Please submit your revised manuscript by Jun 16 2024 11:59PM. If you will need more time than this to complete your revisions, please reply to this message or contact the journal office at [plosone@plos.org](mailto:plosone@plos.org). When you're ready to submit your revision, log on to <https://www.editorialmanager.com/pone/> and select the 'Submissions Needing Revision' folder to locate your manuscript file.

Please include the following items when submitting your revised manuscript:

- A rebuttal letter that responds to each point raised by the academic editor and reviewer(s). You should upload this letter as a separate file labeled 'Response to Reviewers'.
- A marked-up copy of your manuscript that highlights changes made to the original version. You should upload this as a separate file labeled 'Revised Manuscript with Track Changes'.
- An unmarked version of your revised paper without tracked changes. You should upload this as a separate file labeled 'Manuscript'.

If you would like to make changes to your financial disclosure, please include your updated statement in your cover letter. Guidelines for resubmitting your figure files are available below the reviewer comments at the end of this letter.

If applicable, we recommend that you deposit your laboratory protocols in protocols.io to enhance the reproducibility of your results. Protocols.io assigns your protocol its own identifier (DOI) so that it can be cited independently in the future. For instructions see: <https://journals.plos.org/plosone/s/submission-guidelines#loc-laboratory-protocols>. Additionally, PLOS ONE offers an option for publishing peer-reviewed Lab Protocol articles, which describe protocols hosted on protocols.io. Read more information on sharing protocols at <https://plos.org/protocols?utm_medium=editorial-email&utm_source=authorletters&utm_campaign=protocols>.

We look forward to receiving your revised manuscript.

Kind regards,

Peter Wenner
Academic Editor
PLOS ONE

Journal Requirements:

When submitting your revision, we need you to address these additional requirements.

1. Please ensure that your manuscript meets PLOS ONE's style requirements, including those for file naming. The PLOS ONE style templates can be found at 
<https://journals.plos.org/plosone/s/file?id=wjVg/PLOSOne_formatting_sample_main_body.pdf> and 
<https://journals.plos.org/plosone/s/file?id=ba62/PLOSOne_formatting_sample_title_authors_affiliations.pdf>

2. Thank you for stating the following financial disclosure: 
"The study was supported by the German Research Foundation (Deutsche Forschungsgemeinschaft, <https://www.dfg.de/en/research-funding/funding-opportunities>), grant project 397846571 (RR) and FOR 2690 (MS, RR) and SFB 1158 (RC, MS)."

Please state what role the funders took in the study.  If the funders had no role, please state: "The funders had no role in study design, data collection and analysis, decision to publish, or preparation of the manuscript." 
If this statement is not correct you must amend it as needed. 
Please include this amended Role of Funder statement in your cover letter; we will change the online submission form on your behalf.

3. In the online submission form, you indicated that [The data underlying the results presented in the study are available from the authors upon request.]. 
All PLOS journals now require all data underlying the findings described in their manuscript to be freely available to other researchers, either 1. In a public repository, 2. Within the manuscript itself, or 3. Uploaded as supplementary information.
This policy applies to all data except where public deposition would breach compliance with the protocol approved by your research ethics board. If your data cannot be made publicly available for ethical or legal reasons (e.g., public availability would compromise patient privacy), please explain your reasons on resubmission and your exemption request will be escalated for approval.

4. We note that you have included the phrase “data not shown” in your manuscript. Unfortunately, this does not meet our data sharing requirements. PLOS does not permit references to inaccessible data. We require that authors provide all relevant data within the paper, Supporting Information files, or in an acceptable, public repository. Please add a citation to support this phrase or upload the data that corresponds with these findings to a stable repository (such as Figshare or Dryad) and provide and URLs, DOIs, or accession numbers that may be used to access these data. Or, if the data are not a core part of the research being presented in your study, we ask that you remove the phrase that refers to these data.

5. We note that S1 and S2 Figures in your submission contain copyrighted images. All PLOS content is published under the Creative Commons Attribution License (CC BY 4.0), which means that the manuscript, images, and Supporting Information files will be freely available online, and any third party is permitted to access, download, copy, distribute, and use these materials in any way, even commercially, with proper attribution. For more information, see our copyright guidelines: <http://journals.plos.org/plosone/s/licenses-and-copyright>.
 
We require you to either (1) present written permission from the copyright holder to publish these figures specifically under the CC BY 4.0 license, or (2) remove the figures from your submission:
 
a. You may seek permission from the original copyright holder of S1 and S2 Figures to publish the content specifically under the CC BY 4.0 license. 
 
We recommend that you contact the original copyright holder with the Content Permission Form (<http://journals.plos.org/plosone/s/file?id=7c09/content-permission-form.pdf>) and the following text:
“I request permission for the open-access journal PLOS ONE to publish XXX under the Creative Commons Attribution License (CCAL) CC BY 4.0 (<http://creativecommons.org/licenses/by/4.0/>). Please be aware that this license allows unrestricted use and distribution, even commercially, by third parties. Please reply and provide explicit written permission to publish XXX under a CC BY license and complete the attached form.”
 
Please upload the completed Content Permission Form or other proof of granted permissions as an "Other" file with your submission. 
 
In the figure caption of the copyrighted figure, please include the following text: “Reprinted from [ref] under a CC BY license, with permission from [name of publisher], original copyright [original copyright year].”
 
b. If you are unable to obtain permission from the original copyright holder to publish these figures under the CC BY 4.0 license or if the copyright holder’s requirements are incompatible with the CC BY 4.0 license, please either i) remove the figure or ii) supply a replacement figure that complies with the CC BY 4.0 license. Please check copyright information on all replacement figures and update the figure caption with source information. If applicable, please specify in the figure caption text when a figure is similar but not identical to the original image and is therefore for illustrative purposes only.

[Note: HTML markup is below. Please do not edit.]

Reviewers' comments:

Reviewer's Responses to Questions

**Comments to the Author**

1. Is the manuscript technically sound, and do the data support the conclusions?

The manuscript must describe a technically sound piece of scientific research with data that supports the conclusions. Experiments must have been conducted rigorously, with appropriate controls, replication, and sample sizes. The conclusions must be drawn appropriately based on the data presented.

Reviewer #1: Yes

Reviewer #2: Partly

2. Has the statistical analysis been performed appropriately and rigorously?

Reviewer #1: I Don't Know

Reviewer #2: Yes

3. Have the authors made all data underlying the findings in their manuscript fully available?

The [PLOS Data policy](http://www.plosone.org/static/policies.action#sharing) requires authors to make all data underlying the findings described in their manuscript fully available without restriction, with rare exception (please refer to the Data Availability Statement in the manuscript PDF file). The data should be provided as part of the manuscript or its supporting information, or deposited to a public repository. For example, in addition to summary statistics, the data points behind means, medians and variance measures should be available. If there are restrictions on publicly sharing data—e.g. participant privacy or use of data from a third party—those must be specified.

Reviewer #1: No

Reviewer #2: Yes

4. Is the manuscript presented in an intelligible fashion and written in standard English?

PLOS ONE does not copyedit accepted manuscripts, so the language in submitted articles must be clear, correct, and unambiguous. Any typographical or grammatical errors should be corrected at revision, so please note any specific errors here.

Reviewer #1: Yes

Reviewer #2: Yes

5. Review Comments to the Author

Please use the space provided to explain your answers to the questions above. You may also include additional comments for the author, including concerns about dual publication, research ethics, or publication ethics. (Please upload your review as an attachment if it exceeds 20,000 characters)

Reviewer #1: The paper by Sohns et al. investigated the effects of GABA in the periphery, on rodent neurons and on humans. The work on rodent neurons slightly extends existing knowledge, the human experiments represent the novelty of the paper.

**Major:**

**- The experiments were not preregistered as a trial, and therefore the protocol and number of subjects not laid down in a public database before the investigation. Nevertheless, the experiments are approved by an ethics committee, which requires to provide hypotheses, endpoints, and a power calculations. None of this is provided in the manuscript. This should be explicitly mentioned for every question, and clearly distinguish preset and adequately powered hypotheses and those which are exploratory and might have a less favorable type I and II error.
This includes the experimental timeline, which is not easy to understand. E.g. who of the subjects in Fig. S2 proceeded from B to C, and who from B to D. A flowchart might help making this more accessible**
Correct, the study was approved by an ethics committee prior to onset, which did not categorize the study as “clinical study” but handled it according to the Medical Devices Act and the Medical Device Regulation (MDR), therefore not requiring a preregistration with protocol number and number of subjects placed on a public database. Hypotheses, endpoints and power of calculation have been provided in *Statistics* of the revised manuscript.

A flowchart for each experimental protocol has been made and is attached. It should be made clear that not all participants were involved in all trials, there was some overlap but not 100%. We hope that this makes the outlined experiments and the subjects involved more easy to follow. Of note, for the purposes of the outcome measures, it was not mandatory whether subject 1 participated in both flare responses to GABA and histamine itch recordings.

**- “The inability of GABA to generate action potentials in DRG neurons is also consistent with the lack of sensations accompanying intradermal GABA observed here and reported previously (27)”. Although the pain directly induced by GABA might be minimal, and this might explain why modulation of evoked please was investigated: Please provide data on the pain caused by GABA vs. control injection, in particularly given the failure of the only published human study cited (citation 27) to do so.**

We acknowledge the reviewers comment and have added the pain NRS values recorded upon GABA vs. control injection in the *Results* section.

**Minor:
- Figure 3a: In case the SD is negative, the data are probably skewed. The authors need to decide whether non-parametric visualization is more suitable.**

The SD is not negative, it is merely the representation of the SD in one direction – we decided to show the positive SD for one and the other as negative to improve clarity in the Figure. If needed, a SD can be represented in both directions (positive and negative).

**- Line 187: ‘to less than 10% of the initial fluorescence ‘should rather be to less than 10% increase above the initial …**

Amended.

**- Independent repetitions of cellular experiments should be mentioned, in addition to the cell count. E.g. in line 358, assuming 100 cells in a field of view and a 20% response rate to GABA, 19 cells might well be a single run of the experiment, which I would consider highly questionable.**

The number of dishes for the pooled data was amended to the figure legend of Figure 1.

**- Rundown of GABA responses with butemetanide cannot be judged without an identical protocol without this substance.**

In an independent experiment, we examined repetitive applications of GABA in a ten minute interval, in addition to a longer exposure of GABA of five minutes, which reduced the wash time between applications to five minutes. Here, we saw no rundown of the GABA responses in comparison to the experiments in Figure 1H, which we took to mean that Bumetanide was indeed having the expected effect of blocking NKCC1 from restoring the intracellular chloride concentration.

**- Line 277: Why where experiments single and not double blinded?**

Unfortunately, a lack of manpower (experimenters) contributed to the decision to make a single-blinded study. It should be mentioned, that in this case, a bias in outcomes is even less likely, since the experimenter was aware of what was being injected and still no significant effect was evident. Thus, perhaps this is even beneficial for the robustness of the results.

**- Many values are provided with a precision which is probably not justified by the measurement, and a digit less might be the better.**

Agreed, mean values are presented with 1 digit, and only in case exact p-values are reported a maximum of 3 digits is shown.

**- Fig 1B. Is the concentration response constructed only from protocols like in Fig 1A, which has, despite tachyphylaxis in other panels, starts with a high concentration**

Yes, the concentration response followed this protocol exclusively. Actually, we would suggest that the tachyphylaxis observed in Figure 1H, for example, was exclusively due to the combination with bumetanide and in no other panels is there an obvious repetition-induced reduction in GABA response. However, it is possible that the increasing concentrations would blur a possible tachyphylaxis. The protocol followed in the dose response had a wash period of five minutes, similar to that in the bumetanide experiments and it is clear that the increasing concentrations also increases the calcium influx. In fact, the second 200µM is larger than the first, therefore we do not expect an influence of repetitive GABA.

**- Fig 1I. Legend mentions 145 mM KCl, which is probably 45 mM as mentioned in the methods**

Amended.

**- Fig 1J) A Venn diagram seems more suited to visualize the populations than a bar chart**

We displayed a Venn diagram, prior to the bar representation in the Figure of the current submission, however we did not feel it added any valuable representation. We have attached it here for your viewing.

**- Fig 2 C,D,E: Is a scale up to 12 reasonable for a measure in the range 0-10?**

Amended to maximum scale up to 10.

**- Fig 3B: Why did half of the skin blood flow increase in the furosemide pretreated spots occur before the iontophoresis?**

Furosemide itself caused a dose-dependent skin blood flow increase (depicted in Fig 3B) and as iontophoresis was performed 1 min after the injection of furosemide, the increased skin blood flow in the furosemide pretreated spots before iontophoresis (now Fig 3C) is most likely attributed to this. Notably, injection of the more potent NKCC1 blocker torasemide evoked a pronounced flare response and itch and thus secondary effects of loop-diuretics injected into human skin cannot be excluded. We added this point to the *Discussion*. These experiments with torasemide were pilot experiments using intradermal injections of 100μl containing 0.05mg and 0.5mg torasemide (Torem®). The compound evoked pronounced itch and there was development of a wheal and axon reflex flare, all indicative of mast cell degranulation. In confirmation of this, torasemide responses in the skin were blocked by the H1 histamine receptor antagonist cetirizine (oral 25mg, Zyrtec®) in n=2 subjects. We therefore did not consider this pharmakon for further studies (torasemide data not shown) and opted instead for furosemide (Lasix®, 10mg/ml) to examine the effect of NKCC1 blockade on axon-reflex flare, itch and pain.

Reviewer #2: This manuscript addresses the question of whether modulation of cutaneous terminals of primary somatosensory neurons by GABA alters human responses to stimuli that evoke mild to moderate pain or itch. This is a question of potential clinical importance as peripheral GABA signaling might provide opportunities to treat pain and itch caused by peripheral insults. Using dissociated mouse DRG neurons and Ca-imaging, the authors replicate previous studies indicating that GABA depolarizes DRG neuron somata sufficiently to increase intracellular Ca2+ that depends upon a high concentration of intracellular Cl- maintained by NKCC1 pump activity, and that GABA sensitivity correlates with TTX insensitivity (the sensory neurons more likely to be nociceptors or pruriceptors). They then describe various experiments with human volunteers to determine whether excitatory responses to GABA injected into the skin alter neurogenic inflammation, pain, or itch (apparently for the first time, as no references to previous studies involving cutaneous injection of GABA are cited). The most important finding is that GABA injection failed to produce pain or itch, and that injection of neither GABA nor furosemide (which inhibits NKCC1 to reduce intracellular Cl- concentration and hyperpolarize the Cl- equilibrium potential, ECl) altered the volunteers’ ratings of pain or itch induced by other cutaneous stimuli. While these are noteworthy findings and the methods are solid, I have concerns about how the experimental design and results are interpreted, which should be considered when revising the manuscript.

**1. The manuscript stresses the “contrast” and “surprising” difference between the observations of GABA-evoked Ca2+ transients in dissociated mouse DRG neurons and the lack of GABA effects upon injection into human skin. Ignored completely are well-established principles about how a ligand such as GABA can both depolarize a neuron if membrane potential is below the reversal potential for Cl- and produce a net inhibitory effect via a decrease in input resistance (Rin) and shunting of excitatory currents. In nociceptive and pruriceptive DRG neurons, ECl is often depolarized relative to resting membrane potential (RMP) but hyperpolarized relative to AP threshold. For example, if a dissociated soma or peripheral process has an RMP of -65 mV, ECl of -40 mV, and an AP threshold of -35 mV (all of which have been reported for DRG neuron somata), opening of Cl- channels by themselves will not be able to depolarize RMP to the AP threshold. In addition, the increase in Cl- conductance will reduce Rin, decreasing the amplitude of sensory generator potentials and spontaneous depolarizing fluctuations of RMP, and the Cl-mediated depolarization that approaches (but cannot exceed) ECl will inactivate voltage-gated Na+ channels. Thus, there is no reason for surprise at the lack of human GABA-evoked pain and itch when extrapolating from the observations of GABA-evoked Ca2+ transients in dissociated DRG neuron somata.**

We completely agree with the referee and are embarrassed that we did not include the GABA effects of “clamping” membrane potential above the AP threshold and increasing shunting via reduced membrane resistance. We have included those aspects as crucial parts in the *Discussion*.

**2. The fact that the Ca2+ transients are relatively small suggests that the GABA-evoked depolarizations are not large enough to activate a substantial fraction of voltage-gated Ca channels and thus that these depolarizations usually fail to exceed AP threshold. A major limitation of the manuscript is that no electrophysiological measurements were made, so none of these potentials (RMP, ECl, AP threshold, GABA-evoked depolarization relative to AP threshold) are actually known. Of course, these values are not yet obtainable from the cutaneous terminals of rodent or human DRG neurons, but the inhibitory principles are clear and should be discussed as part of the experimental design and interpretation of the results.**

Again, we completely agree with the referee. We have revised the *Discussion* to include the limitations of our imaging approach and specifically the inhibitory principles induced by GABA and how they can contribute to the observed results.

**3. Similarly, the results do not really suggest that “pharmacological intervention of GABA_A_R signaling in human skin is not … promising.” This is because the study is mainly designed to test interventions that target depolarizing effects of GABA. The work of Gamper, Du and colleagues has provided strong evidence that, at least in the DRG, even when depolarizing a nociceptor, GABA has strong inhibitory effects. In principle, the same mechanisms could operate on conduction through peripheral branch points and on the effectiveness of generator potentials in terminals.**

Correct, we have rephrased the therapeutic implications and included the inhibitory GABA effect on the nocicepetive transmission reported by Gamper and colleagues [1].

**4. This study did not examine conditions that might optimize inhibitory effects of GABA in cutaneous receptive fields. The closest it came to testing this idea was in the experiments of Fig.2D,E, examining the effects of furosemide pretreatment and GABA injection on pain evoked by electrical stimulation. There was a lack of apparent analgesic effects from GABA, but this might be because the electrical test stimuli produced only modest pain that was further attenuated by the injection volume effect seen in all the human experiments in this manuscript. Furthermore, the effectiveness of peripheral injections of GABA may be limited by desensitization of GABA receptors, and by potentially rapid uptake and degradation of injected GABA within the skin.**

We agree with the referee and have expanded on the limitations of our in-vivo approach.

**5. Because no pro-algesic effects of GABA were found in this study (Figures 2, 3), the furosemide experiments (expected to reduce the depolarizing effects of GABA) provide very limited information. However, furosemide should enhance the inhibitory effects of GABA, so the cited study reporting itch reduction by furosemide (line736) supports the possibility that enhancement of GABA effects might be therapeutically useful. Again, it would have been useful also to explicitly test whether peripheral GABA injection can have inhibitory effects on pain or itch, and perhaps to test the effects of blocking transport of Cl- out of the cells (in addition to their use of furosemide to block transport of Cl- into the cells).**

We agree with the referee and amended the *Results* on GABA injection evoked pain scores (see also comment above Reviewer#1). Potential effects of peripheral GABA injection on pain was tested and depicted in Figure 2 B/C. To further explore the effects by for instance KCC2 outward Cl- transport blockade is an appreciated suggestion, but we are not aware of a compound that might be suitable for injection into humans.

MINOR COMMENTS
**Line 53 – Furosemide should reduce the outwardly directed Cl- gradient, but brief applications of GABA should mainly reveal the effects of this gradient reduction, not by itself cause “depletion” of the gradient.**

We changed the sentence in the *Abstract* accordingly.

**Line 141 – poly-L-lysine**

Amended.

**Line 191 – what is the duration of each GABA application?**

30 seconds – has been amended in the script.

**Line 352 – why show a weak effect of Ni2+ (n=47, p=0.037) rather than the strong effect mentioned from the highly specific T-type VGCC blocker TTA-P2?**

In contrast to previous evidence that TTA-P2 blockade is reversible [2] we experienced difficulty washing the substance out, with a reduction in calcium transient to GABA even after five minutes, which, in accordance with the previous results that GABA induced calcium transients are stable at a five minute interval, cannot be attributed to a repetitive stimulation. Therefore, we chose to display the evidence for Nickel as we could not be sure that the effect was solely calcium channel blockade. This is similar to the data shown for Conotoxin MVIIC, which both are attached below for your viewing (see Additional Figure 1).

**Line 360 – the important effect is inhibiting transport of Cl- out of the cell to change the Cl- gradient, not the small current mediated by NKCC1 transporters.**

Agreed, the sentence has been changed as suggested.

**Lines 414-418 – the responses should be higher in the neurons with a lower, not higher, TTX sensitivity, if I understand this experiment correctly. Also, which traces are being referred to?**

The interpretation is correct, the details have been amended in the manuscript and we apologize the confusion.

**Line 436 – electrically evoked calcium signals (not “electrical calcium signals”)**

Amended.

**Line 704 – a reference is needed for this statement about mast cell degranulation**

The degranulation of mast cells by codeine was shown before by Church and colleagues [3]. However, in the revised version of the manuscript we omitted this part as it did not provide additional information to our concluding statement that “our chemical and electrical tests in human skin did not include the relevant GABAAR-positive nociceptor class”.

**Line 729 – the observation that human DRG neurons have a more hyperpolarized ECl than rodent DRG neurons should be mentioned (Zhang et al., PMID: 26415765).**

Agreed, species differences of DRG neurons observed by Zhang and colleagues [4] have been mentioned as limitation.

6. PLOS authors have the option to publish the peer review history of their article ([what does this mean?](https://journals.plos.org/plosone/s/editorial-and-peer-review-process#loc-peer-review-history)). If published, this will include your full peer review and any attached files.


If you choose “no”, your identity will remain anonymous but your review may still be made public.


**Do you want your identity to be public for this peer review?** For information about this choice, including consent withdrawal, please see our [Privacy Policy](https://www.plos.org/privacy-policy).

Reviewer #1: No

Reviewer #2: **Yes:** Edgar T. Walters

[NOTE: If reviewer comments were submitted as an attachment file, they will be attached to this email and accessible via the submission site. Please log into your account, locate the manuscript record, and check for the action link "View Attachments". If this link does not appear, there are no attachment files.]

While revising your submission, please upload your figure files to the Preflight Analysis and Conversion Engine (PACE) digital diagnostic tool, <https://pacev2.apexcovantage.com/>. PACE helps ensure that figures meet PLOS requirements. To use PACE, you must first register as a user. Registration is free. Then, login and navigate to the UPLOAD tab, where you will find detailed instructions on how to use the tool. If you encounter any issues or have any questions when using PACE, please email PLOS at [figures@plos.org](mailto:figures@plos.org). Please note that Supporting Information files do not need this step.

*In compliance with data protection regulations, you may request that we remove your personal registration details at any time.* [*(Remove my information/details)*](https://www.editorialmanager.com/pone/login.asp?a=r)*. Please contact the publication office if you have any questions.*

Additional Figures

Add. Figure 1 -- TTA-P2 blockade and Conotoxin MVIIC:

Shows the T-type channel blockade with NiCl2 & TTA-P2 as well as the N-type channel blockade with Conotoxin MVIIC and the difficulty of regaining a responses after the latter two.

Figure 1 -- Version with Venn Diagramm:

Supplementary Figure 1. Generated with PowerPoint.

**S1 Fig Experimental protocol as a flowchart to assess the effect of NKCC1 blockade on GABA-induced responses**

A) GABA or NaCl was injected into each respective forearm and axon-reflex flare (skin vasodilation) recorded using LDI for 10 minutes. Corresponding to figure 2A.
B) Electrically-evoked pain responses to 1Hz half period sinusoidal current and 4Hz sinusoidal current were assessed before and 1 minute after injection in verum and control treated forearms. Corresponding to figure 2B.
C) Furosemide (1mg) or NaCl were injected intradermally in either arm. Corresponding to figure 2C.
D) At baseline (prior to any injections) and 25 minutes after furosemide / NaCl injection, pain ratings in response to continuous 4Hz sinusoidal current and 1Hz half period sinusoidal current were assessed. Corresponding to figure 2D.
E) GABA (1mM) was injected at the same site of previous furosemide and NaCl injection (randomized forearms) and pain responses to 1Hz half period sinusoidal (1mA) and 4Hz sinusoidal (0.2mA) currents were again assessed at 1 minute and 5 minutes after GABA injection. Corresponding to figure 2E.

Supplementary Figure 2. Generated with PowerPoint.

S2 Fig Experimental protocol for the assessment of NKCC1 blockade and chemically-induced itch

A) Skin sites approximately 10 cm distal to the cubital fossa on the left and right arms were injected with either 100µl furosemide or NaCl (randomized left or right). One minute after injection, histamine was applied by iontophoresis at the site of injection. Itch ratings (NRS) were recorded. Corresponding to Figure 3A.
B) Skin sites approximately 10 cm distal to the cubital fossa on the left and right arms were injected with either 100µl furosemide at two different concentrations (0.1mg, light blue or 1mg, dark blue) or NaCl (0.9%). Skin vasodilation (LDI) was recorded. Corresponding to Figure 3B.
C) One minute after injection of Furosemide or NaCl, histamine or codeine was applied by iontophoresis at the site of injection. Skin vasodilation (LDI) was recorded every minute for 10 minutes. 24 hours later, the same protocol was used for assessing the other (histamine or codeine) substance.
D) Pain ratings (NRS) to sinusoidal current at 4Hz at an amplitude of 0.2mA delivered continuously for 1 minute before (baseline) and after intradermal injection of 1mg furosemide (blue diamonds) or 0.9% NaCl (black open circles), recorded every 4 minutes for 36 min (n=9).
E) Pain ratings (NRS) to 1Hz sinusoidal stimulation delivered at current intensities of 0.2, 0.6 and 1mA to the forearm skin sites before injection and 16 and 32 minutes after injection of 1mg Furosemide or NaCl.

1.	Du X, Hao H, Yang Y, Huang S, Wang C, Gigout S, et al. Local GABAergic signaling within sensory ganglia controls peripheral nociceptive transmission. J Clin Invest. 2017;127(5):1741-56. Epub 20170404. doi: 10.1172/JCI86812. PubMed PMID: 28375159; PubMed Central PMCID: PMCPMC5409786.

2.	Choe W, Messinger RB, Leach E, Eckle VS, Obradovic A, Salajegheh R, et al. TTA-P2 is a potent and selective blocker of T-type calcium channels in rat sensory neurons and a novel antinociceptive agent. Mol Pharmacol. 2011;80(5):900-10. Epub 20110805. doi: 10.1124/mol.111.073205. PubMed PMID: 21821734; PubMed Central PMCID: PMCPMC3198916.

3.	Cole ZA, Clough GF, Church MK. Inhibition by glucocorticoids of the mast cell-dependent weal and flare response in human skin in vivo. BrJ Pharmacol. 2001;132(1):286-92.

4.	Zhang XL, Lee KY, Priest BT, Belfer I, Gold MS. Inflammatory mediator-induced modulation of GABAA currents in human sensory neurons. Neuroscience. 2015;310:401-9. Epub 20150928. doi: 10.1016/j.neuroscience.2015.09.048. PubMed PMID: 26415765; PubMed Central PMCID: PMCPMC4633346.
