# Supplementary material for: Depolarization of mouse DRG neurons by GABA does not translate into acute pain or hyperalgesia in healthy human volunteers
Source: PLoS One. 2024 Aug 26;19(8):e0307668. doi: 10.1371/journal.pone.0307668 (PMC11346724; doi:10.1371/journal.pone.0307668)
Supplement: Attachment — Submitted filename: Response_R2.docx [file pone.0307668.s004.docx]

PONE-D-24-10034

Depolarization of mouse DRG neurons by GABA does not translate into acute pain or hyperalgesia in healthy human volunteers

PLOS ONE

Dear Dr. Wenner,

Thank you very much for returning our manuscript to us. We are happy, that the reviewers were satisfied with our comments and edits on the manuscript. It has certainly been improved with the feedback.

Please take this short note as a response, to acknowledge that we have seen the additional comments and editorial advice and have amended them accordingly in the manuscript and within Figure 3 the arrow has been re-added.

Thank you very much.

Best wishes,

Kyra Sohns
